# Temperature and Exciton Concentration Induced Excimer Emission of 4,4′-Bis(4′′-Triphenylsilyl) Phenyl-1,1′-Binaphthalene and Application for Sunlight-Like White Organic Light-Emitting Diodes

**DOI:** 10.1186/s11671-016-1578-3

**Published:** 2016-08-25

**Authors:** Tao Xu, Weiling Li, Xicun Gao, Chang Sun, Guo Chen, Xiaowen Zhang, Chunya Li, Wenqing Zhu, Bin Wei

**Affiliations:** 1 Key Laboratory of Advanced Display and System Applications, Ministry of Education, Shanghai University, 149 Yanchang Road, Shanghai, 200072 People’s Republic of China; 2 School of Petroleum and chemical engineering, Qinzhou University, 12 coastal Avenue, Qinzhou, 535000 People’s Republic of China; 3 Department of Electrical Engineering, Iowa State University, 4565 Union Dr, Ames, IA 50011 USA; 4 Sino-European School of Technology, Shanghai University, 99 Shangda Road, Shanghai, 200444 People’s Republic of China; 5 School of Mechanical & Electrical Engineering, Guilin University of Electronic Technology, 1 Jinji Road, Guilin, 541004 People’s Republic of China

**Keywords:** Sunlight-like WOLEDs, Excimer emission, Monomer emission, Concentration, Electron transportation layer

## Abstract

This paper demonstrates the influence of temperature, exciton concentration, and electron transportation layers on the excimer emission of a novel deep-blue material: 4,4′-bis(4′′-triphenylsilyl) phenyl-1,1′-binaphthalene (SiBN), by studying the photoluminescence and electroluminescence spectra of SiBN-based film. We have further developed sunlight-like and warm-light white organic light-emitting diodes (WOLEDs) with high efficiency and wide-range spectra, using SiBN and bis(2-phenylbenzothiozolato-*N*,C2′)iridium(acetylacetonate) (bt_2_Ir(acac)) as the blue excimer and yellow materials, respectively. The resulting device exhibited an excellent spectra overlap ratio of 82.9 % with sunlight, while the device peak current efficiency, external quantum efficiency, and power efficiency were 18.5 cd/A, 6.34 %, and 11.68 lm/W, respectively, for sunlight-like WOLEDs.

## Background

The developments of organic materials and advanced device structures in organic light-emitting devices (OLEDs) [1, 2] and especially white OLEDs (WOLEDs) [3, 4] have recently attracted much interest for their application in full-color display and next generation energy efficient solid-state lighting [5, 6]. It is known that sunlight is the best lighting source in the world due to its natural properties; therefore, an ideal WOLED should emit a continuous spectrum covering the entire visible range (380–780 nm) and mimic the spectral distribution of natural sunlight [7]. Jou’s group first revealed the concepts of sunlight-style OLED and sunlight spectrum resemblance in 2009 [8] and 2011 [9], respectively. Several methods have been developed to fabricate such WOLEDs: the fabrications of stacked [10] or multilayer [11] OLED structures and the mixture of multi-emissive materials in one emitting layer [12], as well as the use of microcavity for white light emission [13]. However, most of the reported wide-range spectra WOLEDs have used three emissive materials to achieve the precise color balancing, which complicated the device fabrication. Another major challenge of the current WOLEDs is to achieve high device efficiency. Yu et al. demonstrated a sunlight-like WOLED with a high color rendering index of 89, whereas the device efficiencies were lower than 7 cd/A and 3 lm/W [14]. It is thus necessary to develop new approach to fabricate high efficient sunlight-like WOLEDs considering broad range spectra and easy fabrication process, which are applied not only as perfect solar simulators [15–17] but also as an ideal daily lighting source.

To simplify the device fabrication, one approach is to use emissive material known to possess two emissive states: the common monomolecular fluorescence and an additional dimer emission. It is widely known that the excimer states can essentially modify the electronic properties of light-emitting molecular and open up the exceptional possibility to tailor the performance of organic optoelectronic devices [18]. Although the applications of excimer emission have been intensively investigated [19–22], very few reports have discussed the dependences of excimer emission for one specific emissive material in thin films and OLEDs. Recently, our group has reported a novel blue emitter material, 4,4′-bis(4′′triphenylsilyl) phenyl-1,1′-binaphthalene (SiBN) with a binaphthyl backbone modified by (4′′-triphenylsilyl) group [23]. The photoluminescence (PL) spectra of SiBN showed a monomer emission at 415 nm and an excimer emission at 468 nm, which could fully cover the blue region, thus meeting the needs of a wide spectrum in WOLEDs. In addition, the high thermal stability of SiBN was demonstrated, showing a high glass transition temperature at 159.3 °C, which could contribute to the stability and eventual efficiencies of the whole device. As a result, it is worth considering SiBN as a promising candidate for blue emissive material in order to fabricate sunlight-like WOLED with high efficiencies and wide-range spectra.

In this work, we aimed at investigating the influence of temperatures, exciton concentrations, and electron transportation layers (ETLs) on the excimer emissions. Both excimer and monomer emissions were found to depend strongly on the annealing temperature and the doping concentration. The differences in spectra between the SiBN-based OLEDs with different ETLs have been revealed, while the mobility of ETL was found to play an important role in determining the distribution of emission intensity. Furthermore, sunlight-like and warm-light WOLEDs with wide-range spectra, using SiBN as the blue excimer emissive material and bis(2-phenylbenzothiozolato-*N*,C2′)iridium(acetylacetonate)(bt_2_Ir(acac)) as the yellow emissive material, have been developed. The device efficiencies were measured to be 18.5 and 33.0 cd/A for sunlight-like and warm-light WOLEDs, respectively.

## Methods

**Fig. 1:**
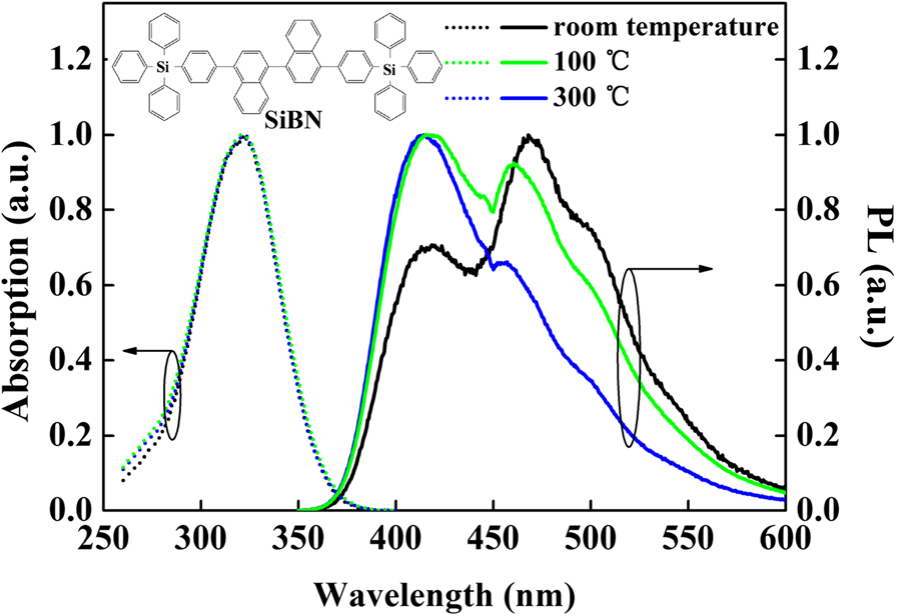
The absorption and PL spectra of SiBN thin films annealing at room temperature (*black*), 100 °C (*green*), and 300 °C (*blue*) for 30 min; the *inset* is the molecule structure of SiBN

We have designed and synthesized a binaphthyl derivative of SiBN modified by (4′′triphenylsilyl) group. The synthesis route of SiBN was described in our previous work [23], while the molecular structure of SiBN is shown in the inset of Fig. 1.

PL spectra of SiBN pristine thin films annealing at different temperatures were measured to investigate the influence of temperature on the excimer emission, while PL and electroluminescence (EL) spectra of OLEDs using SiBN-doped 9,10-di(naphth-2-yl)anthracene (AND) thin films with different concentrations (1, 5, and 20 wt.% for PL; 1 and 5 wt.% for EL) and different ETL layers (TPBi, Bphen, and Bphen: 10 wt.% Cs_2_CO_3_) were obtained. The corresponding OLEDs, referred to as devices A, B, C, and D, were fabricated. The structures of these devices are as follows:

- Device A: ITO/HAT-CN (30 nm)/NPB (10 nm)/AND: 1 wt.% SiBN (20 nm)/TPBi (30 nm)/Liq (1 nm)/Al (100 nm)
- Device B: ITO/HAT-CN (30 nm)/NPB (10 nm)/AND: 5 wt.% SiBN (20 nm)/TPBi (30 nm)/Liq (1 nm)/Al (100 nm)
- Device C: ITO/HAT-CN (30 nm)/NPB (10 nm)/AND: 5 wt.% SiBN (20 nm)/Bphen (30 nm)/Liq (1 nm)/Al (100 nm)
- Device D: ITO/HAT-CN (30 nm)/NPB (10 nm)/AND: 5 wt.% SiBN (20 nm)/Bphen (10 nm)/Bphen: 10 wt.% Cs_2_CO_3_ (20 nm)/Liq (1 nm)/Al (100 nm)

Furthermore, two WOLEDs named devices E and F were fabricated by using SiBN as the blue emissive material and bt_2_Ir(acac) as the yellow emissive material. The structures of devices are as follows:

- Device E: ITO/HAT-CN (30 nm)/NPB (10 nm)/mCP: 8 wt.% bt_2_Ir(acac) (10 nm)/mCP (5 nm)/AND:5 wt.% SiBN (20 nm)/TPBi (30 nm)/Liq (1 nm)/Al (100 nm)
- Device F: ITO/HAT-CN (30 nm)/NPB (10 nm)/TCTA (10 nm)/mCP: 8 wt.% bt_2_Ir(acac) (10 nm)/mCP (5 nm)/AND: 5 wt.% SiBN (20 nm)/TPBi (30 nm)/Liq (1 nm)/Al (100 nm)

In devices, ITO is indium tin oxide; 1,4,5,8,9,11-hexaazatriphenylene-hexacarbonitrile (HAT-CN) was used as the hole injection layer. *N*,*N*′-di(naphthalen-1-yl)-*N*,*N*′)-diphenyl-benzidine (NPB) and 4,4,4-tris (*N*-carbazolyl)-triphenylamine (TCTA) acted as the hole transportation layers (HTLs). 1,3,5-tris(*N*-phenylbenzimiazole-2-yl)benzene (TPBi), 4,7-diphenyl-1,10-phenanthroline (Bphen), and Bphen: 10 wt.% Cs_2_CO_3_ performed as the electron transportation layers. 8-Hydroxyquinolato-lithium (Liq) was electron injection layer, while 1,3-bis(carbazol-9-yl)benzene (mCP) applied as host for yellow phosphor.

All the devices were fabricated in a conventional vacuum (<10^−4^ mbar) chamber by thermal evaporation of organic layers onto a clean glass substrate coated with a 150-nm-thick, ~15 Ω per square ITO layer. Prior to use, the substrate was degreased in an ultrasonic bath by the following sequence: in detergent, de-ionized water, acetone, isopropanol, and then cleaned in a UV-ozone chamber for 15 min. The typical deposition rates, monitored by oscillating quartz, were 0.6 and 5.0 Å/s for organic materials and aluminum (Al), respectively. The device active area which was defined by the overlap between the electrodes was 4 mm^2^ in all cases.

The absorption and PL spectra were measured by a HITACHI F-4500 fluorescence spectrophotometer (Hitachi, Ltd., Tokyo, Japan). The current density-voltage-luminance (*J*-*V*-*L*), current efficiency-luminance-power efficiency (*C*E-*L*-*P*E), and Commission Internationale de L’Eclairage (CIE) were measured by a testing setup consisting of a Keithley 2400 Sourcemeter (Keithley Instruments, Inc., Cleveland, OH, USA) and a Photo Research PR-650 spectrophotometer (Photo Research, Inc., Chatsworth, CA, USA). All the measurements were performed in air at room temperature of 25 °C without device encapsulation.

## Results and Discussion

The absorption and PL spectra of neat SiBN thin films after annealing at 100 and 300 °C have been measured, as it is shown in Fig. 1. When the annealing temperature was increased, the emission at 468 nm originating from excimer emission was decreased, while the monomer emission at 415 nm was enhanced. Such variation can be explained by the intensified movements of electronic cloud existing between SiBN molecules at high annealing temperature, which could lead to the rotation and distortion of the chemical bone, thus lowering the planarization of molecules and weakening the condition of excimer formation. The absorption spectra remained the same at different annealing temperatures, indicating that the molecules could remain stable at high temperature. It should be noted that the decomposition and glass transition temperature of SiBN were as high as 480.7 and 159.3 °C, respectively [23], further indicating that the spectral changes at high annealing temperature were not caused by the thermal degradation of SiBN.

**Fig. 2:**
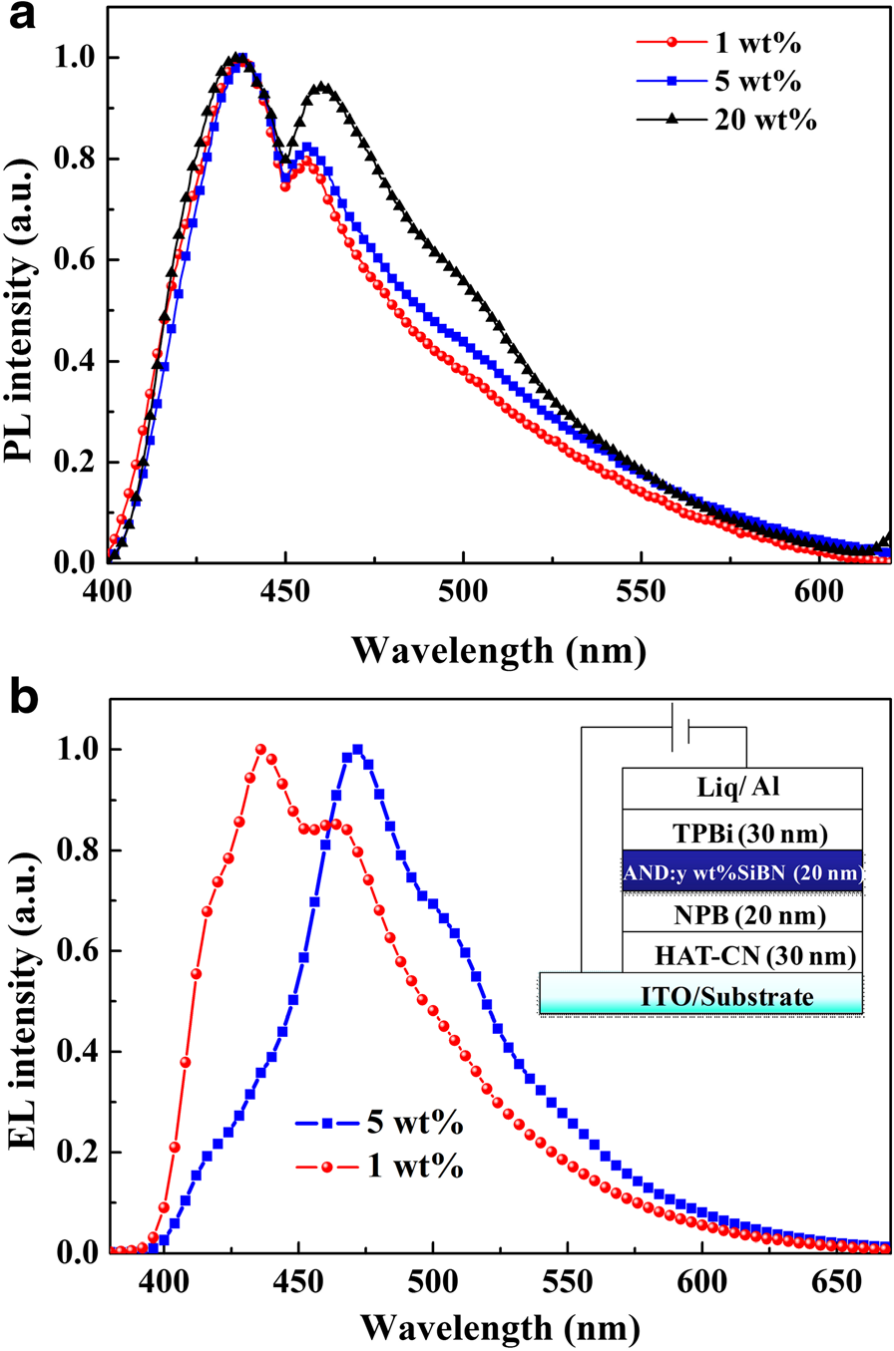
**a** The PL spectra of SiBN-doped AND thin films with various concentrations of 1, 5, and 20 wt.% . **b** The EL spectra of OLEDs using *y* wt.% SiBN-doped AND thin films as the emitting layers (EMLs) (*y* = 1, 5 here)

In PL spectra shown in Fig. 2a, a host-dominated emission of 456 nm shifted to an excimer-dominated emission of 468 nm when the doping concentration was increased to 20 wt.%. Such shift can be attributed to the closer interaction of the active molecules at higher doping concentration, making the formation of excimer excitons easier in such case. The quantitative relative intensities of excimer radiation at 468 nm with respect to emission peaking at 436 nm against the concentration of 1, 5, and 20 wt.% were 63.4, 68.5, and 87.6 %, respectively.

It is widely known that excimers are the molecules in excited state interacting with neighbor non-excited one owing to the intermolecular π–π interactions in single-component organic solids composed of chemically identical molecules, which strongly depends on the doping concentration. Exciplex emission usually occurs at the interface between emissive layer and HTL or ETL as the excited emissive molecule interacting with the inhomogeneous ground-state molecule, while it has no relationship with the doping concentration. In our case, doping-concentration dependence was clearly observed; the emission should thus be originated from excimer [24].

We have investigated the donor emission and acceptor absorption spectra to understand the mechanism of energy transfer, while no overlap was observed between these two spectra, indicating that there is no Förster or Dexter energy transfer from AND to SiBN. In our case, AND was used in the active layer to dilute the concentration of SiBN and prevent concentration quenching. Furthermore, AND has bipolar carrier transport characteristic and can also act as an emitter in WOLED to complete sunlight-like spectrum.

**Fig. 3:**
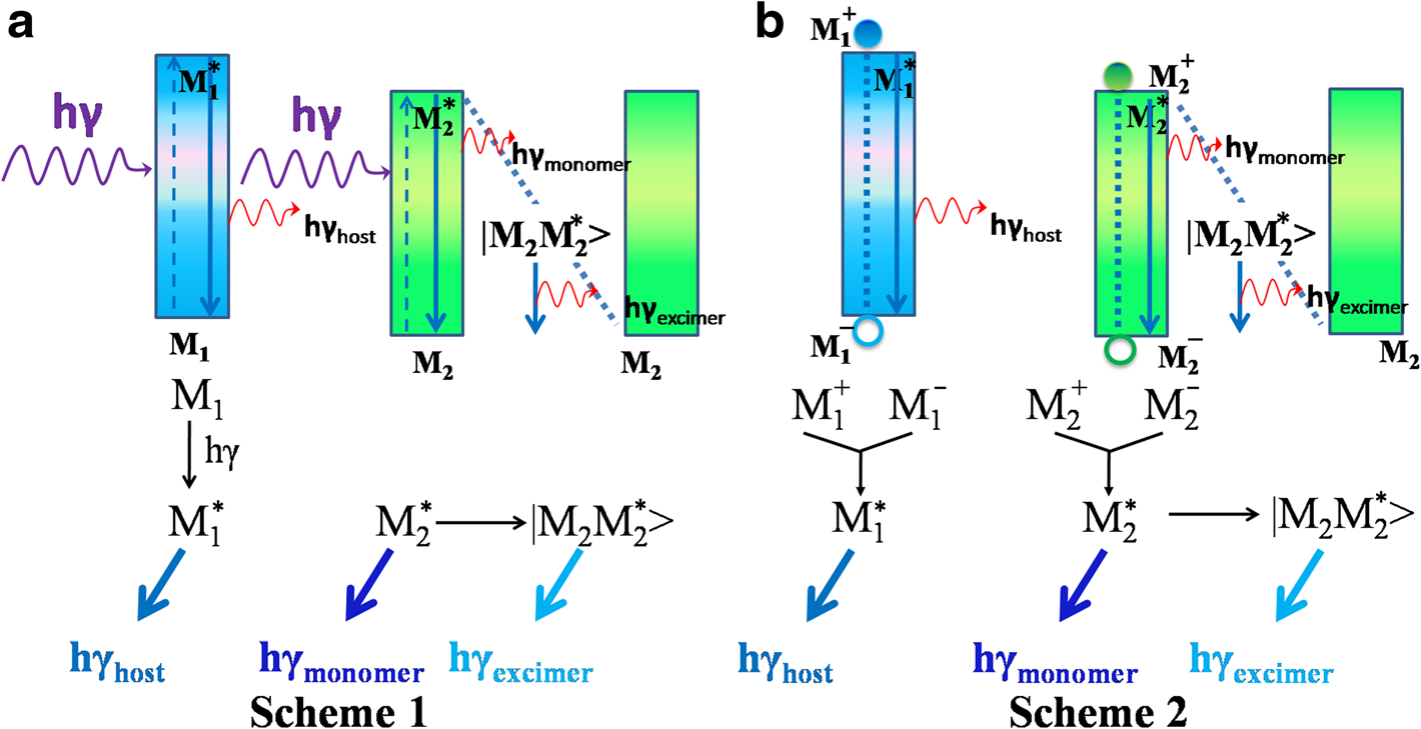
The diagram of exciton formation process in **a** PL and **b** EL of AND:SiBN thin films

Figure 3a describes the photoexcited exciton formation process. The excited state of AND was defined as *M*_1_^*^ which was directly excited from its ground states *M*_1_; and the excited state of SiBN was referred to as *M*_2_^*^, origin from direct photoexcited from its ground states *M*_2_. There were two possible routes for the decay of the excited guest molecules (*M*_2_^*^): monomer emission (at 415 nm) directly generated by radiation and excimer radiation (at 468 nm) generated by the interaction with the ground states of guest molecules. Meanwhile, a fraction of excited excitons (*M*_1_^*^) radiated and generated emission at 436 nm.

Similar results were observed in the EL spectra of OLEDs with different doping concentration, as it is shown in Fig. 2b. Device B with a doping concentration of 5 wt.% exhibited an excimer-dominated emission peaking at 468 nm with a current efficiency of 9.38 cd/A, while device A with a doping concentration of 1 wt.% showed a host-dominated emission peaking at 436 nm, as well as a monomer bulge at 415 nm with a current efficiency of 2.63 cd/A. Such difference between devices A and B can be explained by the weaken SiBN exciton formation at the case of low doping concentration, thus lowering the possibility of excimer formation and inducing the more competitive monomer and host emission.

In EL, as it is shown in Fig. 3b, carriers injected from cathode and anode can be trapped by AND and SiBN directly, generating positive and negative ions (*M*_1_^*+*^, *M*_1_^*−*^, *M*_2_^*+*^, *M*_2_^*−*^). Their recombination contributed to the formation of *M*_1_^*^ and *M*_2_^*^, leading to radiated monomer and host emission. The excited guest molecules could interact with the ground states of guest molecules (*M*_2_) and induced excimer emission.

**Fig. 4:**
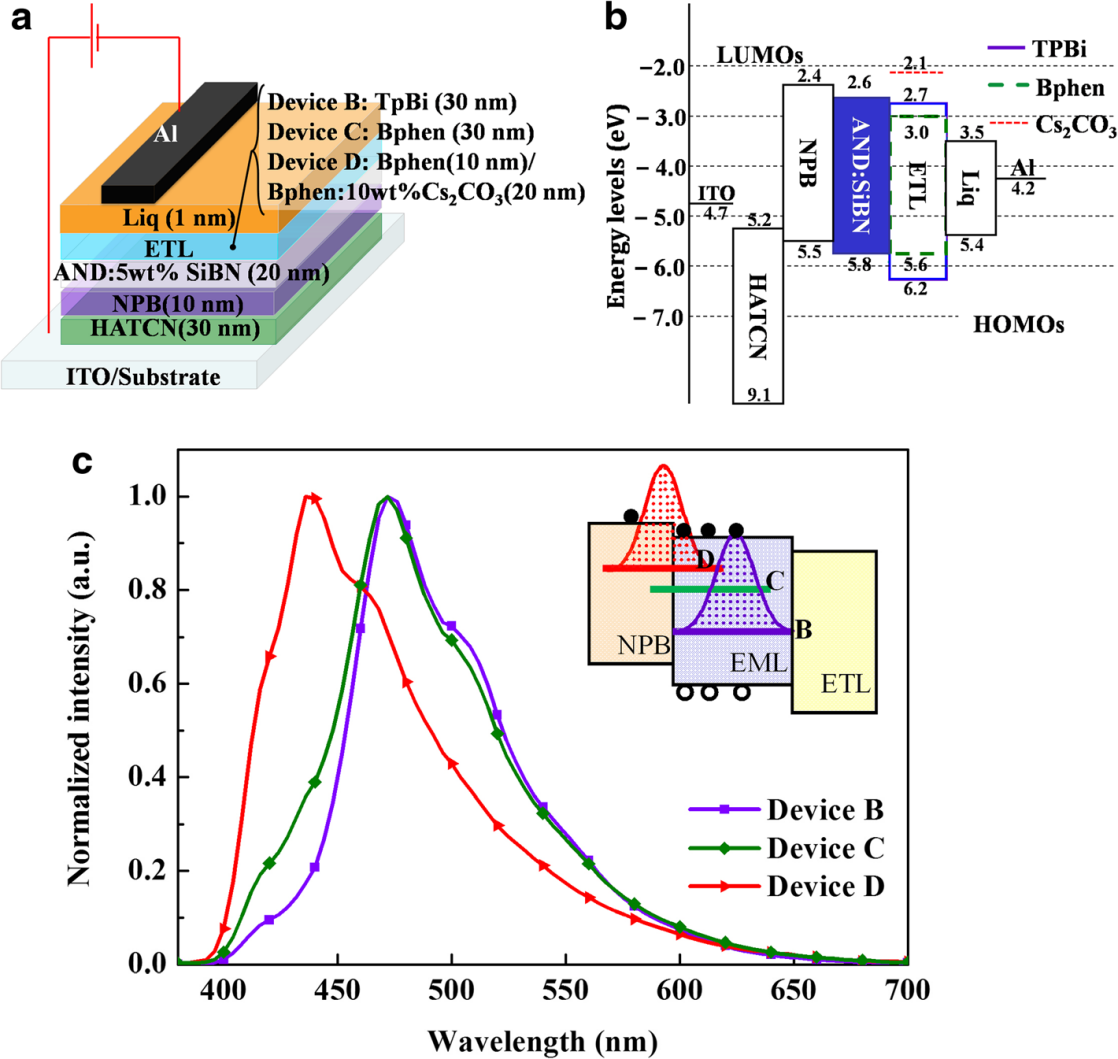
**a** The architecture of OLEDs with various ETLs is ITO/HAT-CN (30 nm)/NPB (10 nm)/AND: 5 wt.% SiBN (20 nm)/ETLs (30 nm)/Liq (1 nm)/Al. **b** The energy diagrams of the devices. **c** EL spectra of the devices. The *inset* is the schematic diagrams of exciton recombination zone

Several ETLs such as TPBi, Bphen, and Bphen: 10 wt.% Cs_2_CO_3_ were applied in the devices with a certain doping concentration to improve the performance of OLED. The structures and energy level diagrams of these devices are shown in Fig. 4a, b. The spectrum of device D employing Bphen: 10 wt.% Cs_2_CO_3_ as the ETL peaked at 436 nm, while the spectra of the rest devices exhibited a peak at 468 nm, as it is shown in Fig. 4c.

In this case, we found that the electron mobility of the ETLs played an important role in determining the emission intensity distribution. Electron mobility of the different ETLs was measured by developing charge-only devices. As a result, Bphen: 10 wt.% Cs_2_CO_3_ had the highest electron mobility in the three ETLs, followed by Bphen and TPBi. Higher mobility of the ETL resulted in a recombination zone closer to the HTL. The recombination zones of the devices with different ETLs were represented as different color bars (purple for device B, green for device C, and red for device D) in the inset of Fig. 4c. A fraction of excitons formed in NPB layer of device D could be easily relaxed. In addition, the chemically active ions, such as Cs^+^ from ETL, were likely to quench the excitons. Caused by the energy relaxation in transportation layer and excitons quenching in emitting layer (EML), the exciton concentration of device D in EML was reduced significantly. As a result, it was difficult to form excimer for device D using ETL with high mobility, resulting in the domination of host emission. Conversely, the relatively low mobility of TPBi (device B) and Bphen (device C) could induce more charge accumulation in the EML and resulted in a stronger excimer emission. The maximum current efficiencies and external quantum efficiency (EQE) of devices B, C, and D were 9.38 cd/A (4.66 %), 6.30 cd/A (3.47 %), and 2.12 cd/A (1.68 %), respectively.

**Fig. 5:**
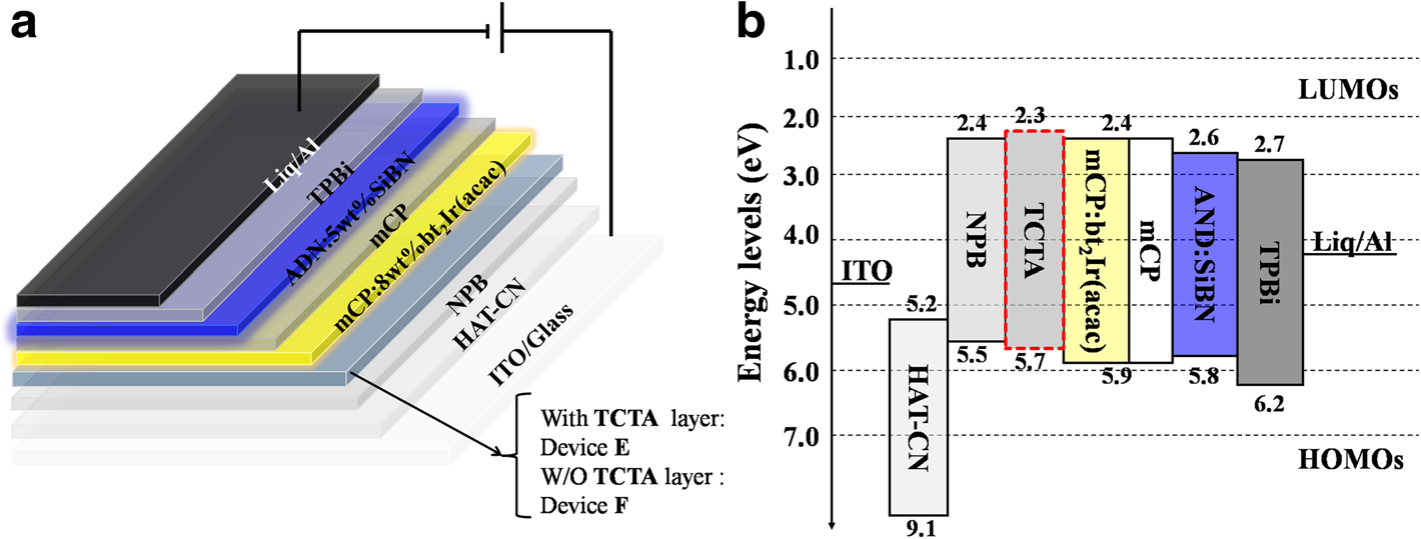
**a** The architectures and **b** the energy diagrams of WOLEDs. The architectures of devices E and F are ITO/HAT-CN (30 nm)/NPB (10 nm)/W/O or with TCTA (10 nm)/mCP: 8 wt.% bt_2_Ir(acac) (10 nm)/mCP (5 nm)/AND: 5 wt.% SiBN (20 nm)/TPBi (30 nm)/Liq (1 nm)/Al

In the EL spectra of SiBN-doped AND thin film, there were three peaks at 415, 436, and 468 nm from monomer emission, host emission, and excimer emission, respectively. The continuous spectrum covering the whole blue region due to the impressive excimer emission motivated us to pursue for WOLEDs using SiBN-doped AND thin film as the blue EML, while TPBi was used as the ETL in this case. Figure 5a, b illustrates the structure and the energy level diagrams of devices E and F, which indicated the excellent energy matching between different materials, thus facilitating carrier injection and transportation [25].

**Fig. 6:**
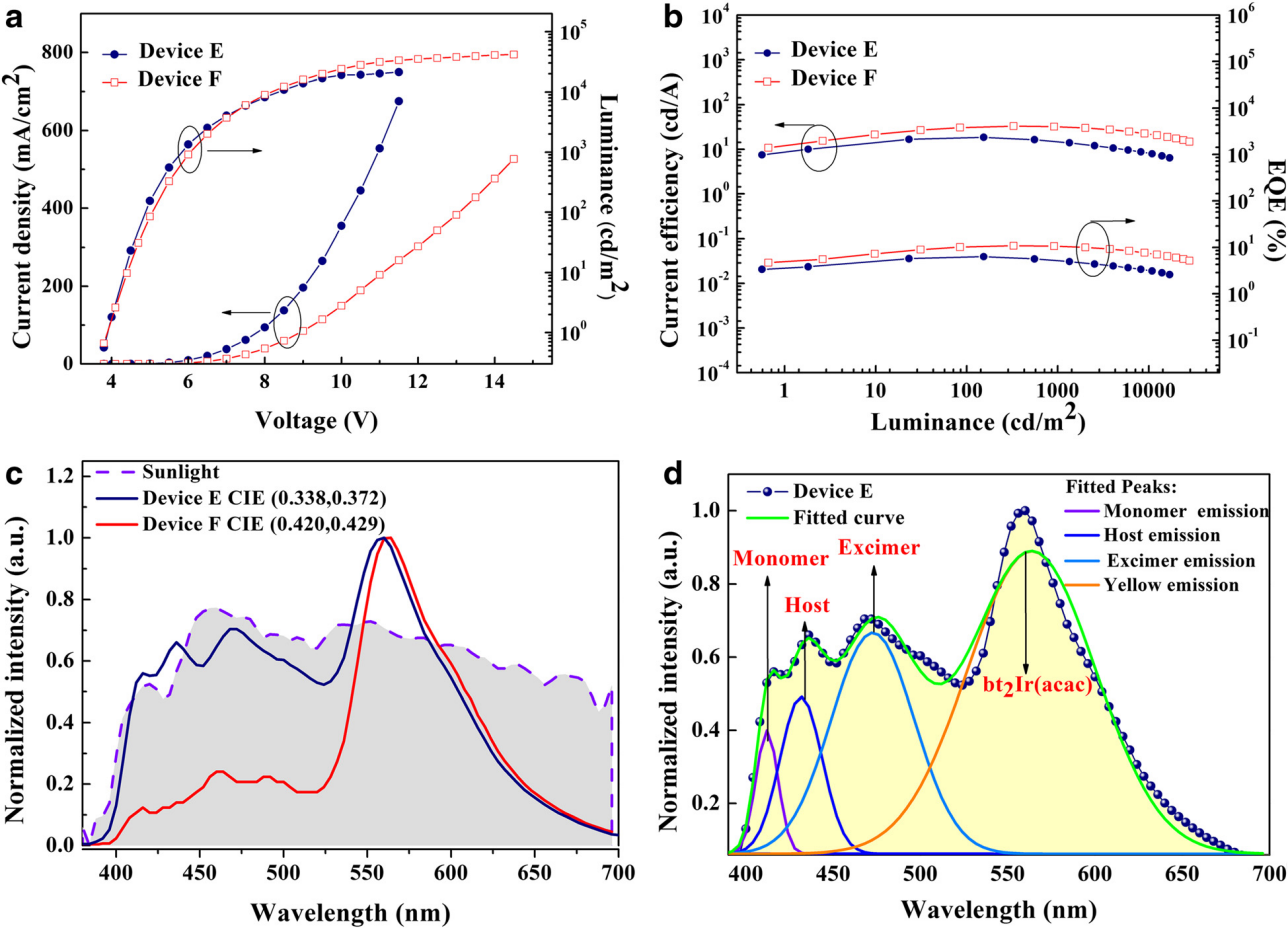
**a** Characteristics of *J-V-L* for devices E (*blue solid circle line*) and F (*red open square line*). **b** *C*E-*L*-EQE curves of devices E (*blue solid circle line*) and F (*red open square line*). **c** EL spectra of devices E and F and the spectrum of sunlight measured by PR650. *Blue line* for device E, *red line* for device F, and *violet dash line* for sunlight. **d** Gauss multi-peaks fitting for the spectra of device E

Efficient and sunlight-like white emission has been achieved in this work. Figure 6a plotted the current density-voltage characteristic curves of devices E and F. Device F showed lower current density and higher luminance than those of device E. Devices E and F achieved maximum luminance values of 41673.6 cd/m^2^ at 14.5 V and 21254.4 cd/m^2^ at 11.5 V, respectively.

Figure 6b reveals the *C*E-*L*-EQE properties. Device E showed the maximum current efficiency and EQE of 18.5 cd/A and 6.34 %, while the maximum values of device F were 33.0 cd/A and 10.92 %. In addition to the interesting blue emission mechanism mentioned above, the origin of the yellow phosphorescent emission can be attributed to the recombination of the partial injected electrons in yellow EML (mCP: 8 wt.% Ir(ppy)_3_), as well as the following conversion into photons via relaxation of both singlet and triplet excitons. As shown in Fig. 6c, device E presented a broad range spectrum with a CIE of (0.338, 0.372) and its spectrum curve almost fitted that of sunlight, exhibiting a spectra overlap ratio (the overlap area of the spectra between the WOLED and measured sunlight from 380 to 700 nm divide the integral area of sunlight) of 82.9 %, making it an appropriate candidate for sunlight-like illumination. The color rendering index of the sunlight is 89, which is an impressive value for di-chromatic WOLEDs. The composition of the EL spectrum of device E was analyzed by Gauss multi-peaks fitting. As it is shown in Fig. 6d, the measured EL spectrum is consistent with the fitted curve which could be decomposed into four peaks consisting of the spectral of monomer, host, excimer, and yellow emitter. Device F had better performance on both current efficiency and EQE than device E, but the spectrum of device F exhibited a warmer white sharing a CIE of (0.399, 0.435). Such performance can be ascribed to the stronger emission of the yellow phosphorescent emissive layer, and it opens up a way to obtain color-tunable WOLEDs.

The differences in the EL spectra between these devices can be attributed to the addition of TCTA, which was widely considered as a material not only to help facilitate holes transportation but also to block electrons. Therefore, carriers could be further confined in emissive layer [26]. As it is shown in Fig. 5b, holes must overcome a barrier of 0.4 eV in order to enter mCP for device E, whereas this value decreased to 0.2 eV at the interface of NPB/TCTA for device F, allowing holes to easily penetrate into TCTA layer and to reach EML. Furthermore, the triplet level of TCTA is higher than that of NPB, which could reduce the outflow of excitons in EML, thus enhancing the proportion of yellow triplet excitons [27]. As a result, the current efficiency, power efficiency, and EQE of device F have been improved comparing to these of device E by 78.4, 65.5, and 72.2 %, respectively, at the cost of lower blue emission shown in Fig. 6c.

## Conclusions

In conclusion, we have investigated the influence of temperature, concentration, and ETL on monomer and excimer emission of SiBN, as well as the application for fabricating sunlight-like WOLEDs. We found that the PL emission intensity of excimer at 468 nm reduced with the increasing annealing temperature due to the decreased planarization degree of SiBN molecules, thus weakening the intermolecular aggregation. In addition, the PL and EL emissions of SiBN doped in AND exhibited that devices with a high doping concentration produced relatively stronger excimer emission compared to those with low concentration. The EL spectra of SiBN-based electroluminescent device with different ETL revealed that intensity of excimer emission was increased with ETL in a lower mobility, resulting from the enhanced concentration of excitons in EMLs. Finally, we have developed sunlight-like WOLEDs with high efficiency and wide-range spectrum, by using SiBN and bt_2_Ir(acac) as the blue excimer and yellow materials, respectively. The developments of such sunlight-like WOLED with two emissive materials can not only simplify the fabrication process but also meet the needs of a healthy lighting source.

## Abbreviations

*C*E*-L-P*E: Current efficiency-luminance-power efficiency
CIE: Commission Internationale de L'Eclairage
EL: Electroluminescence
EML: Emitting layer
EQE: External quantum efficiency
ETL: Electron transportation layer
HTL: Hole transportation layer
*J*-*V*-*L*: Current density-voltage-luminance
OLED: Organic light-emitting diode
PL: Photoluminescence
WOLED: White organic light-emitting diode
